# High cognitive reserve postpones dementia onset in older adults with cardiometabolic disease

**DOI:** 10.1002/alz.090983

**Published:** 2025-01-09

**Authors:** Abigail Dove, Wenzhe Yang, Serhiy Dekhtyar, Jie Guo, Jiao Wang, Anna Marseglia, Davide Liborio Vetrano, Rachel A. Whitmer, Weili Xu

**Affiliations:** ^1^ Aging Research Center, Karolinska Institutet, Stockholm Sweden; ^2^ Tianjin Medical University, Tianjin China; ^3^ Division of Clinical Geriatrics, Center for Alzheimer Research, Department of Neurobiology, Care Sciences and Society, Karolinska Institutet, Stockholm Sweden; ^4^ Aging Research Center, Karolinska Institutet and Stockholm University, Stockholm Sweden; ^5^ University of California, Davis School of Medicine, Sacramento, CA USA

## Abstract

**Background:**

Cardiometabolic diseases (CMDs) including type 2 diabetes, heart disease, and stroke have been linked to increased dementia risk. We examined whether high levels of cognitive reserve (CR) are associated with an attenuation in the CMD‐dementia association and lower levels of CMD‐related brain pathologies.

**Method:**

Within the UK Biobank, 216,178 dementia‐free participants aged ≥60 were followed for up to 15 years for incident dementia via linage to medical records. At baseline, CMDs were ascertained from medical records and self‐reported medical history. Latent class analysis was used to generate an indicator of cognitive reserve (CR; low, moderate, and high) based on education, occupational attainment, confiding in others, social contact, leisure activities, and television watching time. A subsample (n = 13,663) underwent brain magnetic resonance imaging (MRI) scans 9 years after baseline. Volumes of total gray matter (GMV), hippocampus (HV), and white matter hyperintensities (WMHV) were ascertained, and mean diffusivity (MD) and fractional anisotropy (FA) in white matter tracts were collected through diffusion tensor imaging.

**Result:**

CMDs were associated with 57% increased risk of dementia (HR 1.57 [95% CI 1.48, 1.67]). In joint effect analysis, dementia risk was 35% lower among people with CMDs and moderate‐to‐high CR (HR 1.78 [1.66, 1.91]) compared to low CR (HR 2.13 [1.97, 2.30]) (reference: CMD‐free, moderate‐to‐high CR). Having moderate‐to‐high compared to low CR significantly attenuated the association between CMDs and lower GMV and HV.

**Conclusion:**

Moderate‐to‐high cognitive reserve may partially mitigate CMD‐related dementia risk. Resistance to neurodegeneration may account for this alleviating effect.

